# Targeting CD39 in combination with IL-2/anti-IL-2 complexes enhances cytotoxic immunity and limits tumor progression

**DOI:** 10.3389/fimmu.2026.1730342

**Published:** 2026-01-26

**Authors:** Carolina Abrate, Valentina Brunotto, Sabrina N. Bossio, Santiago Boccardo, Jimena Tosello Boari, Pamela Caudana, Lara Hernandez, Camila Gimenez, Martin G. Theumer, Christine Sedlik, Maria C. Amezcua-Vesely, Simon C. Robson, Adriana Gruppi, Eliane Piaggio, Eva V. Acosta Rodríguez, Carolina L. Montes

**Affiliations:** 1 Departamento de Bioquímica Clínica, Facultad de Ciencias Químicas, Universidad Nacional de Córdoba, Córdoba, Argentina; 2 Centro de Investigaciones en Bioquímica Clínica e Inmunología (CIBICI-CONICET), Córdoba, Argentina; 3 INSERM U932 Immunity and Cancer, Department of Translational Research, PSL University, Institut Curie Research Center, Paris, France; 4 Center for Inflammation Research, Departments of Anesthesia and Medicine, Beth Israel Deaconess Medical Center, Harvard Medical School, Boston, MA, United States

**Keywords:** CD39, CD8^+^ T cells, exhaustion, IL-2/anti-IL-2 complexes, immunotherapy

## Abstract

Immunotherapies revolutionized cancer treatment, yet their efficacy remains constrained by the tumor’s immunosuppressive microenvironment. Here, we evaluated whether combining CD39 blockade with other modalities of immunotherapy such as IL-2/anti-IL-2 complexes (IL-2cx) administration could further enhance T cell-mediated antitumor responses and improve tumor control. We demonstrated that CD39 deficiency in MC38 tumor-bearing CD39KO (Entpd1 null) mice decreases tumor growth. This better tumor growth control was associated with increased infiltration of PD-1^High^ CD8^+^ T cells, expressing elevated levels of exhaustion markers and transcription factors such as TOX. This PD-1^High^ CD8^+^ T cell subset also exhibited a higher frequency of IFN-γ-producing and cytotoxic (Granzyme B^+^, Perforin^+^) cells. In contrast, the less immunogenic B16F10-OVA model did not show significant differences in tumor growth; however, CD39KO mice displayed an increased frequency of antigen-specific, pre-exhausted (PD-1^Int^) CD8^+^ T cells, a population recognized as a key target of immunotherapy. Pharmacological CD39 blockade with POM-1, when combined with IL-2cx treatment to redirect IL-2 activity, enhanced the accumulation of pre-exhausted CD8^+^ T cells with cytotoxic potential, thereby improving tumor control. This combinatorial strategy also reshaped the tumor immune landscape by increasing activated NK cells, elevating Granzyme B expression in CD4^+^ T cells, and decreasing immunosuppressive M-MDSCs expressing CD39, CD38, and CD73. Collectively, our findings demonstrate that integrating purinergic pathway inhibition with IL-2–based immunotherapies can coordinately reprogram lymphoid and myeloid compartments, attenuate immunosuppressive mechanisms within the tumor microenvironment, and amplify antitumor immunity, providing a strong rationale for advancing this strategy toward clinical translation.

## Introduction

Immune checkpoint blockade (ICB) therapies have marked a turning point in cancer treatment. Nevertheless, a considerable proportion of patients either fail to respond or eventually develop resistance over time (1, 2). The clinical efficacy of these strategies remains limited, in part, by the immunosuppressive nature of the tumor microenvironment (TME). Tumors actively shape their microenvironment to suppress antitumor immunity, creating a niche that limits effector immune cell infiltration, activation, and function (3). This immunosuppressive context is maintained through multiple mechanisms, including chronic antigen stimulation, metabolic stress, hypoxia, and the accumulation of immunoregulatory molecules and cell types (4).

CD8^+^ T cells are central players in tumor control due to their cytotoxic capacity (5). However, within the TME, these cells often acquire an exhausted phenotype, characterized by impaired cytokine production, reduced proliferation, and persistent expression of CD39 and inhibitory receptors (iRs) such as PD-1, TIM-3, and LAG-3, among other features (6, 7). Exhausted CD8^+^ T cells are not entirely dysfunctional, but their capacity to effectively control tumor growth is impaired, limiting the success of immunotherapy. Notably, the exhausted CD8^+^ T cell compartment is heterogeneous, comprising pre-exhausted and terminally exhausted subsets with distinct functional capacities (8). These different subsets of exhausted CD8^+^ T cells have distinct implications for therapeutic response. Pre-exhausted CD8^+^ T cells retain higher responsiveness to ICB compared with their terminally exhausted counterparts (9), making their preservation and expansion a therapeutic priority.

In this context, CD39 has emerged as a key modulator of T cell dysfunction (10), playing a critical role in shaping the immunosuppressive TME and contributing to the impairment of CD8^+^ T cell responses (11, 12). CD39 is an ectonucleotidase that hydrolyzes extracellular ATP (eATP) to AMP, which is subsequently converted into adenosine by CD73 (13). Elevated eATP levels act as danger signals, driving pro-inflammatory responses and promoting immune cell recruitment through purinergic receptor signaling (14). Once converted to adenosine, however, the balance shifts toward immunosuppression, as this metabolite potently limits CD8^+^ T cell activation, proliferation, and cytokine production (15, 16).

Notably, CD39 is expressed not only by tumor cells but also by various immune cells, including CD8^+^ T cells, Natural Killer (NK) cells, regulatory T cells (Tregs), and myeloid-derived suppressor cells (MDSCs), and the endothelium, all of which contribute to the establishment of an immunosuppressive milieu (17, 18).

In recent years, numerous therapeutic strategies harnessing the immune system have been developed (19), and growing evidence indicates that combining distinct immunotherapeutic approaches can synergistically enhance antitumor efficacy and improve clinical outcomes (20). Within this framework, CD39 has emerged as a promising target due to its central role in modulating immune responses (21). Blockade of CD39, either with monoclonal antibodies (22, 23) or enzymatic inhibitors such as POM-1 (24), has been shown to enhance antitumor immunity and delay tumor progression in preclinical models.

In parallel, IL-2/anti-IL-2 complexes (IL-2cx), which redirect IL-2 activity toward NK and CD8^+^ T cells, have demonstrated the capacity to potentiate antitumor responses by activating NK cells, reinvigorating exhausted CD8^+^ T cells, and broadening tumor-specific T cell responses (25). These distinct approaches seem to engage complementary mechanisms of action, suggesting that their combination could provide superior therapeutic benefit.

Building on this rationale, we first analyzed the impact of CD39 deficiency in two tumor models. In MC38 tumor-bearing mice, CD39 absence enhanced tumor growth control, whereas in the B16F10-OVA model, this did not significantly impact tumor progression, although it fostered the accumulation of pre-exhausted OVA-specific CD8^+^ T cells within the TME. Based on these observations, we next evaluated whether combining CD39 blockade with IL-2cx therapy could further overcome resistance in this setting. Indeed, the combined treatment further improved tumor control by enhancing CD8^+^ T cell effector function and reshaping the immune landscape toward a less immunosuppressive state.

## Materials and methods

### Mouse

C57BL/6 wild-type (WT) and CD39 knockout (CD39KO) mice (6 to 10 weeks) were housed at the animal facility of the CIBICI-CONICET/Facultad de Ciencias Quimicas-UNC. All animal studies were conducted in compliance with institutional guidelines and approved by the Institutional Animal Care and Use Committee of the Facultad de Ciencias Químicas, Universidad Nacional de Córdoba, Argentina (RD-2019-1689-E-UNC-DEC#FCQ, RD-2023-1938-E-UNC-DEC#FCQ). The Entpd1 null mice (CD39KO) mice were kindly provided by Dr. Simon C. Robson (Beth Israel Deaconess Medical Center, Boston MA) (26).

### Cell lines

MC38 was kindly provided by Dr. Clothilde Théry. B16F10-OVA cell line was purchased from ATCC. Both cell lines were maintained in DMEM (GIBCO) supplemented with 10% fetal bovine serum (FBS; Natocor), 1 mM L-glutamine, 25 mM HEPES, and 40 µg/mL gentamicin. In both MC38 and B16F10-OVA tumor-bearing mice, neither CD45+ nor CD45- cells express CD39 within TME after engraftment in CD39KO mice (**Supplementary Figure 1A**), confirming that MC38 and B16F10-OVA tumor cell lines do not express CD39.

### *In vivo* tumor models

Male C57BL/6 or CD39KO mice were anesthetized with ketamine/xylazine (100/10 mg/kg body weight) administered intraperitoneally (i.p.), and subsequently inoculated subcutaneously (s.c.) with either 5 × 10^5^ MC38 cells or 1 × 10^6^ B16F10-OVA cells. Tumors and draining lymph nodes (dLNs) were collected on day 17 post-injection (p.i.). Tumor volume was measured on 7–17 days p.i. using a manual caliper to determine the longest (D) and shortest (d) tumor diameters. Volume was calculated using the formula: tumor volume = (d × D²) × 0.5. Tumors were disaggregated mechanically and enzymatically with 2 mg/mL collagenase IV and 50 U/mL DNase I (Roche). In experiments assessing effector function, tumor-infiltrating mononuclear cells were enriched by density gradient centrifugation using Ficoll-Hypaque or Percoll (GE Healthcare). All procedures were performed following protocols previously established in our laboratory (12, 27).

### Flow cytometry

Single cell suspensions were stained with fluorochrome-conjugated monoclonal antibodies against mouse (fluorochromes and clones, **Supplementary Table S1**). Gating strategies for cellular populations from tumors and dLNs are shown in **Supplementary Figure 1B**.

For *ex vivo* intracellular cytokines, TFs, and CD107a, mononuclear cells were stained using protocols previously established in our laboratory (12, 27).

OVA-specific CD8^+^ T cells were detected with a PE-conjugated OVA^257–264^ H-2Kb dextramer (Immudex) according to the manufacturer´s instructions. Samples were acquired using a BD LSRFortessa flow cytometer and analyzed using FlowJo software.

### POM-1 and IL-2cx treatment

C57BL/6 WT and CD39KO mice were inoculated with B16F10-OVA tumor cells as previously described. Treatment administration began on day 5 p.i., when tumors became palpable. The CD39 inhibitor POM-1 (TOCRIS) was injected i.p. in 200 µL PBS containing 250 µg of POM-1 on days 5, 7, 10, and 13 p.i.

For IL-2cx treatment, mice received daily i.p. injections of 200 µL IL-2cx from day 7 to day 12 p.i. The complex was prepared by mixing 15,000 IU of recombinant human IL-2 (Clinigen Healthcare Ltd) with 4.5 µg of anti-human IL-2 monoclonal antibody (Mab602, Bio-Techne), followed by incubation at 37°C for 30 minutes before injection. For the POM-1/IL-2cx combined treatment, mice received co-injections of both compounds according to the individual schedules used for IL-2cx and POM-1 monotherapies.

### Statistical analysis

GraphPad Prism 9.0.0 software was used for statistical analysis. Prior to statistical testing, data were evaluated for outliers using the ROUT method, which were excluded if identified, and assessed for normality to determine the appropriate statistical test. Samples from two different experimental groups were considered unpaired, whereas samples involving cell populations obtained from the same individual were considered paired. The statistical test used for each comparison is detailed in each figure and was chosen according to the software’s recommendations. P-values ≤0.05 were considered statistically significant.

## Results

### CD39 absence enhances tumor growth control and shapes CD8^+^ T cell responses in MC38 tumor-bearing mice

**Figure 1:**
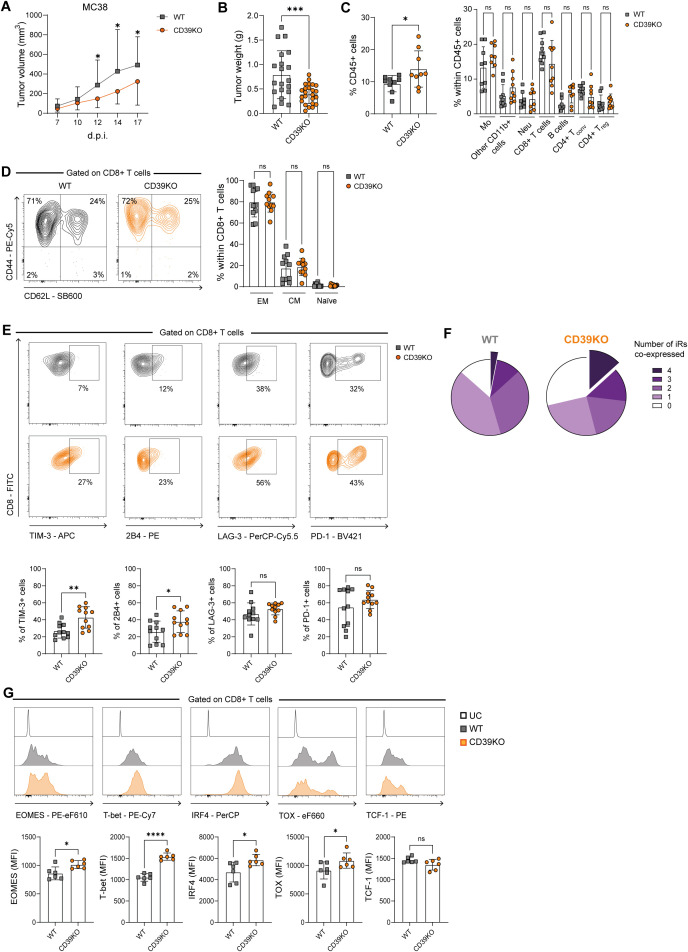
Tumor growth and immune profiling of WT and CD39KO MC38 tumor-bearing mice. WT (gray) and CD39KO (orange) mice were injected s.c. with MC38 tumor cells **(A)**. Tumor volume measured on days 7, 10, 12, 14, and 17d.p.i. **(B)** Tumor weight at 17 d.p.i. **(C)** (Left) Frequency of total CD45^+^ tumor-infiltrating cells. (Right) CD45^+^ immune cell populations. **(D)** Representative contour plots and frequencies of Effector memory (EM) (CD44^+^CD62L^-^), Central memory (CM) (CD44^+^CD62L^+^), and naïve (CD44^-^CD62L^+^) T-I CD8^+^ T cells. **(E)** Representative contour plots and frequencies of T-I CD8^+^ T cells expressing iRs. **(F)** Pie chart shows the proportion of T-I CD8^+^ T cells co-expressing 0 to 4 iRs (n = 11) (TIM-3, 2B4, LAG-3 and PD-1). **(G)** Expression (MFI) of TF (Eomes, T-bet, IRF4, TOX, and TCF-1) in T-I CD8^+^ T cells. **(B-F)** data were analyzed at 17 d.p.i. All results were obtained from at least 2 independent experiments. Data is presented as mean ± SD. Statistical analysis was performed using unpaired Student’s *t*-test **(B, C, E, F)** or one-way ANOVA with Sidak’s multiple comparisons test **(A, C, D)**. ns: not significant; *P ≤ 0.05; **P ≤ 0.01; ***P ≤ 0.001; ****P ≤ 0.0001.

We first evaluated the impact of CD39 deficiency on tumor progression. WT or CD39KO mice were injected with the highly immunogenic MC38 tumor cells. From day 12 post-injection (p.i.) until the experimental endpoint at day 17 p.i., CD39KO mice exhibited significantly reduced tumor volumes compared to their WT counterparts (**Figure 1A**). Consistently, tumor weight at endpoint was also lower in CD39KO mice (**Figure 1B**). Flow cytometric analysis revealed a higher frequency of tumor-infiltrating (T-I) CD45^+^ leukocytes in CD39KO mice than in WT mice (**Figure 1C**). However, no significant differences were observed in the composition of CD45^+^ immune cell subpopulations between the two groups.

Given the critical role of CD8^+^ T cells in tumor control, we next focused on this T-I T cell population. We assessed their activation and differentiation status and found that, in both WT and CD39KO mice, the majority of CD8^+^ T cells exhibited an effector memory (EM) phenotype (CD62L^-^CD44^+^), while central memory (CM) cells (CD62L^+^CD44^+^) accounted for around 20% and naïve cells (CD62L^+^CD44^-^) were nearly absent (**Figure 1D**). Importantly, no significant differences were observed in the relative frequencies of these subsets between groups.

We then analyzed iRs and transcription factors (TFs) associated with CD8^+^ T cell activation and exhaustion. CD39KO mice showed a higher frequency of CD8^+^ T cells expressing TIM-3 and 2B4, but not of LAG-3 and PD-1, compared to WT mice (**Figure 1E**). Additionally, CD39KO mice showed higher frequencies of CD8^+^ T cells co-expressing 4 iRs than WT mice (**Figure 1F**; **Supplementary Figure 1C**). Moreover, T-I CD8^+^ T cells from CD39KO mice displayed elevated expression level (MFI) of the TFs Eomes, T-bet, IRF4, and TOX, while TCF-1 levels were comparable to those in WT mice (**Figure 1G**). Accordingly, CD39KO mice exhibited a higher percentage of TOX-expressing CD8^+^ T cells and similar frequencies of TCF-1^+^ cells compared to WT mice (**Supplementary Figure 1D**).

Together, these results show that CD39 deficiency limits MC38 tumor growth and is associated with increased tumor infiltration by CD8^+^ T cells displaying an exhausted phenotype characterized by elevated expression of multiple iRs and TFs.

### CD39KO mice exhibit increased frequencies of tumor-infiltrating PD-1^High^ CD8^+^ T cells with a cytotoxic phenotype

Since the previous analysis showed that T-I CD8^+^ T cells from CD39KO mice express multiple iRs and exhaustion-associated TFs, we next investigated the heterogeneity within this population, focusing on subsets defined by PD-1 expression levels. A hallmark of T cell exhaustion is its heterogeneity (8), and previous studies have demonstrated that different levels of PD-1 expression can distinguish between subsets of exhausted T cells (28, 29). Specifically, high PD-1 expression characterizes terminally exhausted cells, whereas intermediate expression defines pre-exhausted subsets.

**Figure 2:**
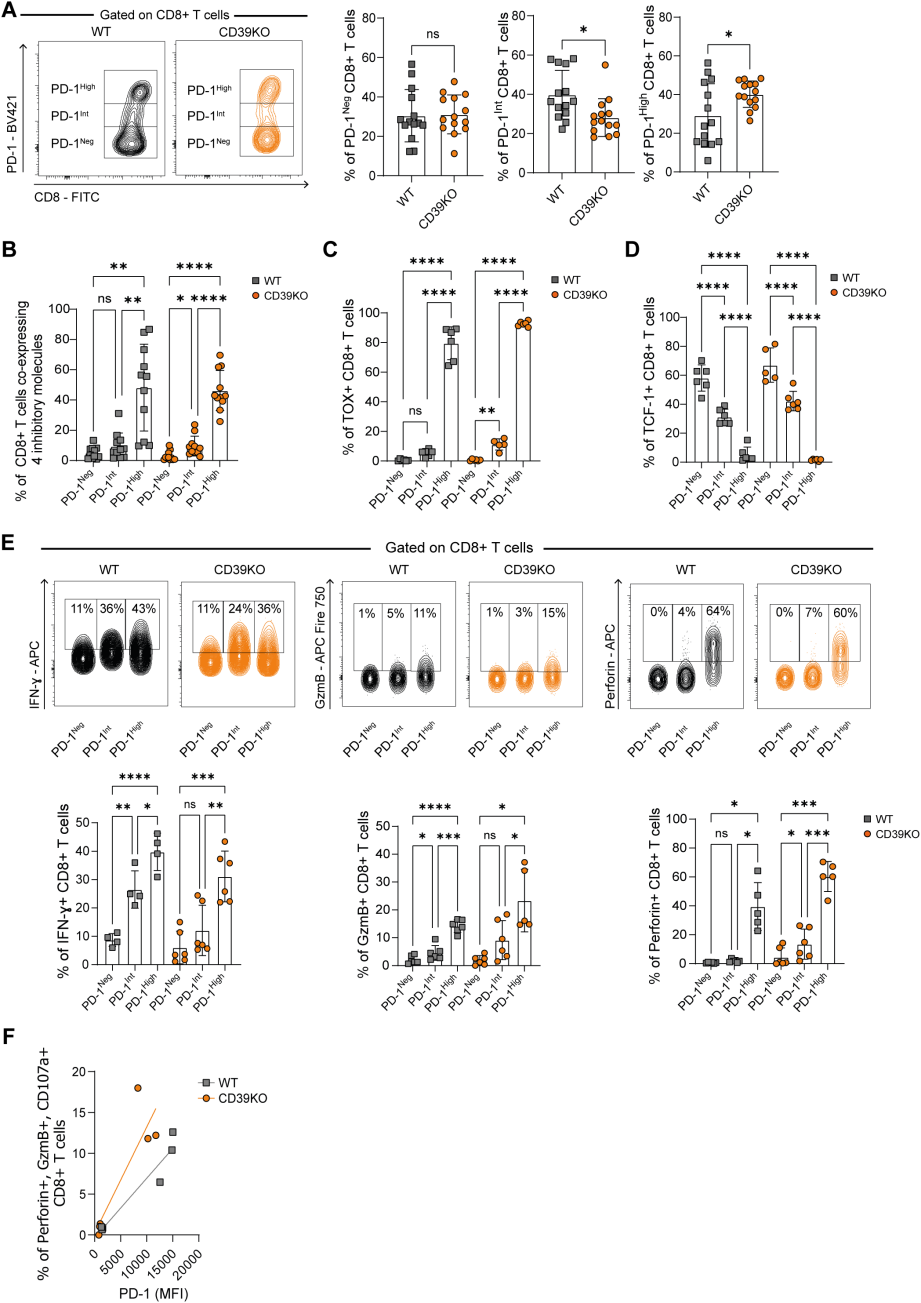
PD-1 expression defines phenotypic and functional heterogeneity in T-I CD8^+^ T cells. WT (gray) and CD39KO (orange) mice were injected s.c. with MC38 tumor cells. **(A)** Representative contour plots and frequencies of T-I PD-1^Neg^, PD-1^Int^, and PD-1^High^ CD8^+^ T cells. **(B)** Frequencies of PD-1^Neg^, PD-1^Int^, and PD-1^High^ CD8^+^ T cells co-expressing 4 inhibitory molecules. **(C)** Frequencies of TOX^+^ cells within PD-1^Neg^, PD-1^Int^, and PD-1^High^ CD8^+^ T cells **(D)**. Frequencies of TCF-1^+^ cells within PD-1^Neg^, PD-1^Int^, and PD-1^High^ CD8^+^ T cells **(E)** Representative contour plots (upper panel) and frequencies (lower panels) of IFN-γ^+^, GzmB^+^, or Perforin^+^ cells within PD-1^Neg^, PD-1^Int^, and PD-1^High^ total T-I CD8^+^ T cells. **(F)** Representative correlation plots showing the relationship between PD-1 expression levels (MFI) and Perforin^+^, GzmB^+^, and CD107a^+^ total T-I CD8^+^ T cells. All data were analyzed on day 17 p.i. All results were obtained from at least 2 independent experiments. Data is presented as mean ± SD. Statistical significance was determined using unpaired Student’s *t*-test **(A)**, or one-way ANOVA with Sidak’s multiple comparisons test **(B–E)**, and Pearson correlation test **(F)**. ns: not significant; *P ≤ 0.05; **P ≤ 0.01; ***P ≤ 0.001; ****P ≤ 0.0001.

To explore this heterogeneity among T-I exhausted CD8^+^ T cells from WT and CD39KO mice, we classified them into PD-1^High^, PD-1^Int^, and PD-1^Neg^ populations. As shown in **Figure 2A**, all three subsets were identified in MC38 tumor-bearing mice of both WT and CD39KO. Comparative analysis revealed that CD39KO mice displayed a significantly higher proportion of PD-1^High^ CD8^+^ T cells and a lower frequency of PD-1^Int^ cells compared to WT mice.

Further analysis of iRs and inhibitory molecules expression revealed that T-I PD-1^High^ CD8^+^ T cells, in WT as well as in CD39KO mice, presented the highest frequencies of cells co-expressing 4 inhibitory molecules (PDL-1, 2B4, TIM-3, and LAG-3), compared to PD-1^Int^ or PD-1^Neg^ populations (**Figure 2B**). In particular, TIM-3, a marker associated with terminal exhaustion (30), was predominantly expressed in the PD-1^High^ population (**Supplementary Figure 2A**).

We also investigated exhaustion-related TFs, including TOX and TCF-1 (31, 32). PD-1^High^ CD8^+^ T cells exhibited the highest frequency and mean fluorescence intensity (MFI) of TOX expression, compared to the other subsets (**Figure 2C**; **Supplementary Figure 2B**). Conversely, TCF-1 expression was largely restricted to PD-1^Int^ and PD-1^Neg^ cells and was almost absent in the PD-1^High^ CD8^+^ T cells (**Figure 2D**; **Supplementary Figure 2C**).

Importantly, we complemented these phenotypic analyses with functional assays. Upon *in vitro* PMA/Ionomycin stimulation, PD-1^High^ cells from both WT and CD39KO mice showed the highest frequency of IFN-γ–producing cells (**Figure 2E**). Additionally, analysis of molecules related to cytotoxic function within T-I CD8^+^ T cell population, revealed that PD-1^High^ population from WT and CD39KO mice presented higher frequencies of Perforin and Granzyme B (GzmB) producing cells than PD-1^Int^ or PD-1^Neg^ CD8^+^ T cells (**Figure 2E**). Importantly, within this PD-1^High^ compartment, CD39 KO mice display higher frequencies of GzmB^+^ and Perforin^+^ CD8^+^ T cells compared to WT mice (**Supplementary Figure 2D**).

Moreover, we observed a positive correlation (WT, r=0.9652 and *P* ≤0.01; CD39KO, r=0.88 and *P* ≤0.05) between PD-1 expression levels and the proportion of polyfunctional CD8^+^ T cells co-expressing CD107a, Perforin, and GzmB (**Figure 2F**).

Collectively, these results indicate that the absence of CD39 favors the accumulation of cytotoxic PD-1^High^ CD8^+^ T cells with features of terminal exhaustion. Furthermore, the enhanced cytotoxic profile of PD-1^High^ CD8^+^ T cells in CD39KO mice is consistent with their improved MC38 tumor control.

### CD39 deficiency does not substantively impact B16F10-OVA growth but increases pre-exhausted CD8^+^ T cell infiltration

To extend our observations from the MC38 model, we next analyzed the impact of CD39 deficiency in the less immunogenic B16F10-OVA melanoma model, which is widely employed to study antigen-specific CD8^+^ T cell responses and provides a model to determine impact of checkpoint blockade on immunogenicity. This approach allowed us to determine whether the absence of CD39 similarly affected tumor growth and to further dissect the dynamics of OVA-specific CD8^+^ T cells within the tumor and draining lymph nodes (dLNs).

**Figure 3:**
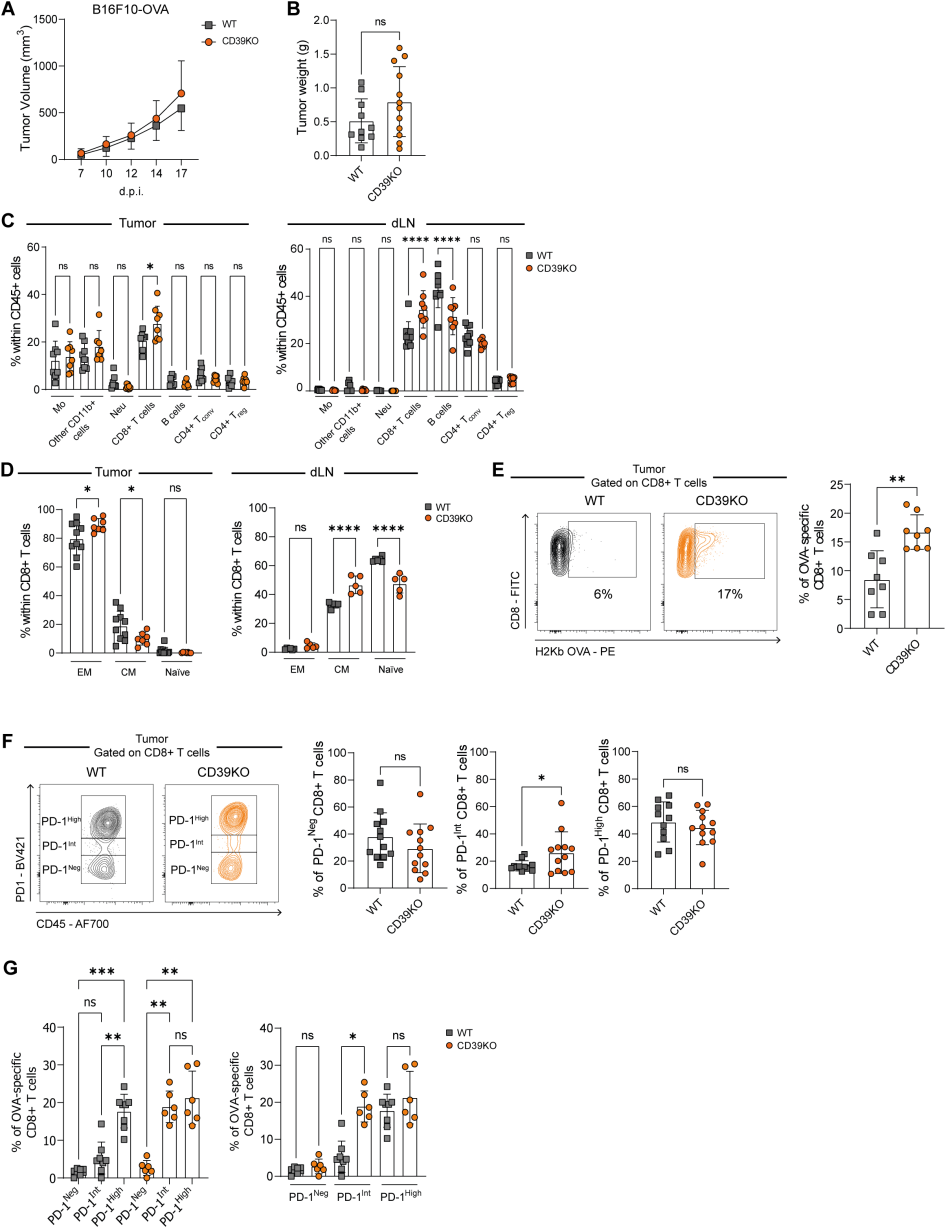
Tumor progression, immune infiltration, and antigen-specific CD8^+^ T cell responses in WT and CD39KO mice bearing B16F10-OVA tumors. WT (gray) and CD39KO (orange) mice were injected s.c. with B16F10-OVA tumor cells **(A)**. Tumor volume measured on days 7, 10, 12, 14, and 17 p.i. **(B)** Tumor weight on day 17 p.i. **(C)** Frequencies of CD45^+^ subpopulations in tumors and dLNs. **(D)** frequencies of effector memory (EM) (CD44^+^CD62L^-^), central memory (CM) (CD44^+^CD62L^+^), and naïve (CD44^-^CD62L^+^) CD8^+^ T cell subsets in tumors and dLNs. **(E)** Representative contour plots and frequencies of T-I OVA-specific CD8^+^ T cells (H-2Kb OVA^+^) **(F)** Representative contour plots and frequencies of T-I PD-1^Neg^, PD-1^Int^, and PD-1^High^ CD8^+^ T cells. **(G)** Frequencies of OVA-specific CD8^+^ T cells within PD-1^Neg^, PD-1^Int^, and PD-1^High^ T cell subsets. **(B-G)** Data were analyzed on day 17 p.i. Results were obtained from at least 2 independent experiments. Data is presented as mean ± SD. Statistical analysis was performed using unpaired Student’s *t*-test **(B, E, F)** or one-way ANOVA with multiple comparisons test **(A, C, D, G)**. ns: not significant; *P ≤ 0.05; **P ≤ 0.01; ****P ≤ 0.0001.

In these studies, using B16F10-OVA, we observed that CD39KO recipient mice exhibited similar tumor growth kinetics and tumor weight at the experimental endpoint (17 days p.i.), when compared to WT mice (**Figures 3A, B**).

Next, we analyzed various immune cell populations in tumors and dLNs. CD39KO mice exhibited higher frequencies of CD8^+^ T cells in both tissues compared to WT mice (**Figure 3C**), accompanied by a relative reduction in B cells in dLNs, with no notable differences in the total frequency of CD45^+^ cells (**Figure 3C**; **Supplementary Figure 3A**).

We then assessed the activation and differentiation status of CD8^+^ T cells in tumors and dLNs using CD44 and CD62L expression. The absence of CD39 promoted the accumulation of EM T-I CD8^+^ T cells at the expense of the CM population (**Figure 3D**). As in MC38 tumors, naïve CD8^+^ T cells were virtually absent. In dLNs, CD39KO mice displayed higher frequencies of CM and lower frequencies of naïve CD8^+^ T cells, with no significant changes in EM cells compared to WT mice.

Analysis of antigen-specific CD8^+^ T cells revealed that CD39KO mice harbored a higher frequency of OVA-specific T-I CD8^+^ T cells compared to WT mice (**Figure 3E**).

We further analyzed the distribution of exhausted CD8^+^ T cell subsets. As observed in the MC38 model, CD8^+^ TILs from both CD39KO and WT B16F10-OVA tumor–bearing mice were classified into PD-1^High^, PD-1^Int^, or PD-1^Neg^ populations based on PD-1 expression levels (**Figure 3F**). Notably, CD39KO mice exhibited a higher frequency of PD-1^Int^ CD8^+^ T cells than WT mice. We also demonstrated that PD-1^High^ CD8^+^ T cells in both groups correspond to terminally exhausted cells, as they co-express multiple iRs, including high levels of TIM-3 (**Supplementary Figures 3B, C**). Accordingly, PD-1^High^ CD8^+^ T cells displayed the highest expression of TOX and the lowest levels of TCF-1, compared to PD-1^Int^ and PD-1^Neg^ subsets (**Supplementary Figures 3D, E**). In line with previous findings (9), terminally exhausted CD8^+^ T cells (PD-1^High^) exhibited the highest frequencies of IFN-γ–producing cells (**Supplementary Figure 3F**) and expression of cytotoxic molecules, including CD107a, GzmB, and Perforin (**Supplementary Figure 3G**).

In contrast, PD-1^Int^ CD8^+^ T cells maintained TCF-1 expression (**Supplementary Figure 3E**) and the ability to produce IFN-γ (**Supplementary Figure 3F**), consistent with a pre-exhausted phenotype.

Interestingly, CD39KO mice showed similar proportions of OVA-specific CD8^+^ T cells within both the pre- and terminally exhausted populations, whereas WT mice exhibited a higher proportion of OVA-specific CD8^+^ T cells among terminally exhausted cells compared to the pre-exhausted and PD-1^Neg^ subsets (**Figure 3G**). Moreover, pre-exhausted OVA-specific CD8^+^ T cells were significantly more abundant in CD39KO mice than in WT mice.

Taken together, these findings demonstrate that, the absence of CD39 promotes the accumulation of effector and tumor antigen-specific CD8^+^ T cells within the TME, although not impacting tumor growth in this specific B16F10-OVA model.

### Blocking CD39 ectonucleotidase activity enhances the antitumor response of IL-2cx therapy

Since our results in the B16F10-OVA model showed that CD39 deficiency favors the accumulation of pre-exhausted CD8^+^ T cells, which are key targets of immune ICB therapy (9), we reasoned that targeting CD39 might synergize with immunotherapies that boost CD8^+^ T cell responses. In this context, IL-2cx redirects IL-2 activity to CD8^+^ T and NK cells and has been shown to enhance antitumor CD8^+^ T cell responses, leading to improved tumor control in B16F10 tumor-bearing mice (25). We therefore evaluated the effect of a combined therapeutic approach, blocking CD39 activity with the pharmacological inhibitor POM-1 together with IL-2cx administration.

**Figure 4:**
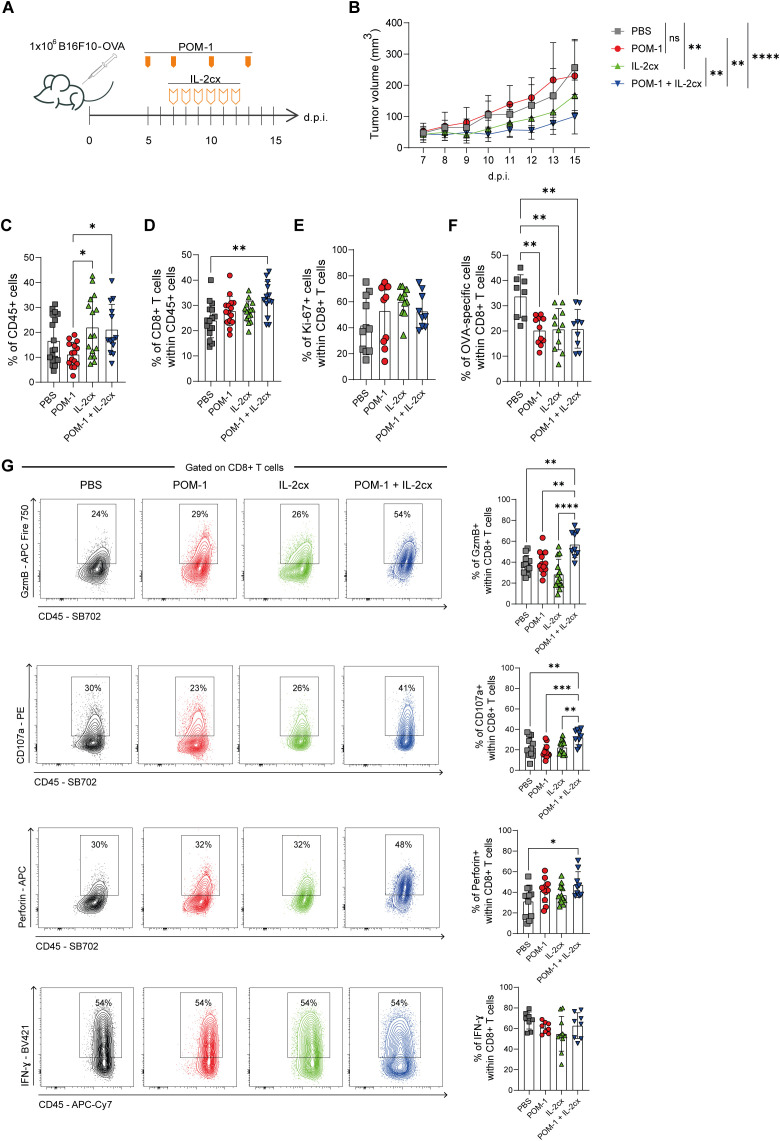
Pharmacological inhibition of CD39 combined with IL-2cx enhances antitumor CD8^+^ T cell response. WT mice were injected s.c. with B16F10-OVA cells and treated with PBS (gray), POM-1 (red), IL-2/anti-IL-2 complex (IL-2cx, green), or the combination POM-1+ IL-2cx (blue). **(A)** Schematic representation of treatment protocol. **(B)** Tumor volumes measured on 7-15 d.p.i. Statistical analysis was performed on day 15 d.p.i. **(C)** Frequencies of T-I CD45^+^ leukocytes. **(D)** Frequencies of T-I CD8^+^ T cells. **(E)** Frequencies of T-I Ki-67^+^ CD8^+^ T cells. **(F)** Frequencies of T-I OVA-specific CD8^+^ T cells. **(G)** Representative contour plots and frequencies of GzmB^+^, CD107a^+^, Perforin^+,^ or IFN-γ^+^ total T-I CD8^+^ T cells. All data were collected on day 15 p.i. Results are representative of 4 independent experiments. Data are presented as mean ± SD. Statistical analysis was performed using one-way ANOVA with multiple comparisons. Non-significant differences are not shown; *P ≤ 0.05; **P ≤ 0.01; ***P ≤ 0.001; ****P ≤ 0.0001.

Following tumor engraftment, B16F10-OVA tumor-bearing WT mice were injected with PBS as control, or treated with POM-1, IL-2cx, the combination of POM-1 and IL-2cx, as depicted in **Figure 4A**. We found that CD39 inhibition with POM-1 alone had no effect on tumor progression compared to the control group (**Figure 4B**). In line with previous reports (25), mice treated with IL-2cx exhibited delayed tumor growth relative to controls. At the experimental endpoint (15 days p.i.), mice receiving the POM-1/IL-2cx combined therapy displayed significantly reduced tumor volume compared to control, POM-1, and IL-2cx groups. Moreover, combination-treated mice showed slower tumor growth, with differences becoming evident by day 12 p.i. compared to all other groups (**Supplementary Figure 4A**).

Analysis of tumor-infiltrating leukocytes showed that mice treated with POM-1 alone exhibited lower frequencies of CD45^+^ cells compared to IL-2cx or the combination, while no other significant differences were detected (**Figure 4C**). Mice treated with the POM-1/IL-2cx combined therapy exhibited higher frequencies of tumor-infiltrating CD8^+^ T cells than the control group (**Figure 4D**). No differences were observed in the frequency of Ki-67^+^ CD8^+^ T cells among the treated groups (**Figure 4E**). When focusing on OVA-specific CD8^+^ T cells, all treated groups (IL-2cx, POM-1, or the combination) showed reduced frequencies compared to PBS controls (**Figure 4F**; **Supplementary Figure 4B**). We also examined PD-1 and TOX expression on OVA-specific CD8^+^ T cells across all treatment groups. More than 80 percent of OVA-specific CD8^+^ T cells displayed high PD-1 expression (PD-1^High^) in all conditions, including PBS controls, and TOX expression showed a similar pattern (**Supplementary Figure 4C**).

We next assessed cytotoxic molecules and found that mice treated with the POM-1/IL-2cx combined therapy exhibited higher frequencies of T-I CD8^+^ T cells expressing GzmB and CD107a compared to all other groups (**Figure 4G**). In addition, the frequency of Perforin^+^ CD8^+^ T cells was increased in the POM-1/IL-2cx combined therapy group relative to the control group. However, no significant differences were observed in the frequencies of IFN-γ–producing CD8^+^ T cells among the groups.

In addition, we analyzed CD8^+^ T cells in dLNs and found that the treatments did not affect the frequency of CD45^+^ cells, CD8^+^ T cells, the expression of cytotoxic molecules, or the proportion of IFN-γ–producing CD8^+^ T cells (**Supplementary Figures 4D, E**). Moreover, none of the treatment strategies resulted in detectable OVA-specific CD8^+^ T cells in the dLNs (data not shown).

Together, these results demonstrate that CD39 inhibition with pharmacological antagonist potentiates the efficacy of IL-2cx therapy, leading to enhanced CD8^+^ T cell infiltration and cytotoxic activity within the TME.

### CD39 inhibition combined with IL-2cx therapy increases cytotoxic molecules expression on pre-exhausted CD8^+^ T cells

**Figure 5:**
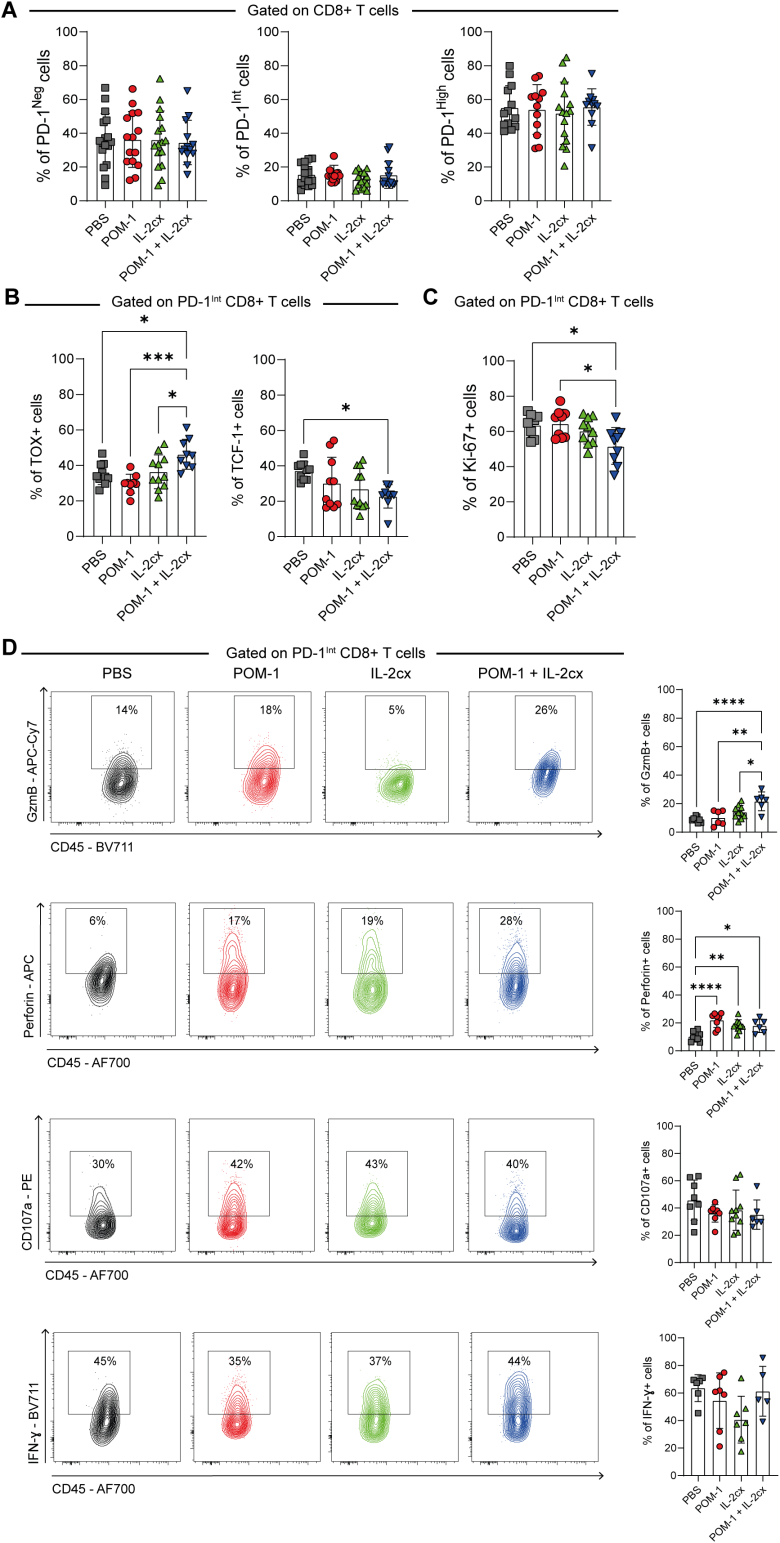
Treatment with POM-1 plus IL-2cx increased the frequency of PD-1^Int^ CD8^+^ T cells with cytotoxic potential. WT mice were injected s.c. with B16F10-OVA cells and treated with PBS (gray), POM-1 (red), IL-2/anti-IL-2 complex (IL-2cx, green), or the combination POM-1 + IL-2cx (blue). **(A)** Frequencies of PD-1^Neg^, PD-1^Int^, and PD-1^High^ total T-I CD8^+^ T cells. **(B)** Frequencies of TOX- expressing (left) and TCF-1^+^ expressing (right) PD-1^Int^ total T-I CD8^+^ T cells. **(C)** Frequencies of Ki-67^+^ expressing PD-1^Int^ total T-I CD8^+^ T cells. **(D)** GzmB^+^, Perforin^+^, CD107a^+^, or IFN-γ^+^ PD-1^Int^ total T-I CD8^+^ T cells. All data were collected on day 15 p.i. Data are presented as mean ± SD. Results are representative of 4 independent experiments. Statistical analysis was performed using one-way ANOVA with multiple comparisons. Non-significant differences are not shown; *P ≤ 0.05; **P ≤ 0.01; ***P ≤ 0.001; ****P ≤ 0.0001.

Because the balance between progenitor and terminally exhausted T cells can influence therapeutic outcomes (9), we next evaluated whether the treatments affected the frequency or functional status of pre-exhausted and terminally exhausted CD8^+^ T cells in B16F10-OVA tumor–bearing mice. To this end, we analyzed the distribution of T-I CD8^+^ T cell subsets defined by PD-1 expression. No significant differences were observed in the frequencies of PD-1^Neg^, PD-1^Int^ or PD-1^High^ CD8^+^ T cells among the treated and control groups (**Figure 5A**). However, we found that POM-1/IL-2cx combined therapy increased the frequency of TOX^+^ cells within the pre-exhausted PD-1^Int^ subset compared to all other groups, while simultaneously reducing the frequency of TCF-1^+^ cells in this population relative to the PBS group (**Figure 5B**). Additionally, PD-1^Int^ CD8^+^ T cells from POM-1/IL-2cx treated group exhibited a reduced proliferation capacity, as evidenced by lower frequencies of Ki-67^+^ cells compared to the POM-1 or PBS-treated group (**Figure 5C**).

Notably, the combined therapy resulted in higher frequencies of GzmB^+^ PD-1^Int^ CD8^+^ T cells compared to the other treatments evaluated (**Figure 5D**). Furthermore, treatment with POM-1, IL-2cx, or their combination increased the % Perforin^+^ PD-1^Int^ CD8^+^ T cells compared to the control group, although no significant differences were observed in the frequencies of CD107a and IFN-γ-expressing cells. Analysis of the PD-1^High^ CD8^+^ T cells showed that the tested treatments did not increase the expression of cytotoxic molecules (GzmB, Perforin, CD107a) or the proportion of IFN-γ-expressing cells (**Supplementary Figure 5A**).

Overall, these data suggest that POM-1/IL-2cx combined treatment modulates the functional profile of pre-exhausted CD8^+^ T cells by increasing GzmB expression, which may contribute to their cytotoxic potential.

### Combined CD39 inhibition and IL-2cx treatment reshapes tumor-infiltrating immune cell populations

**Figure 6:**
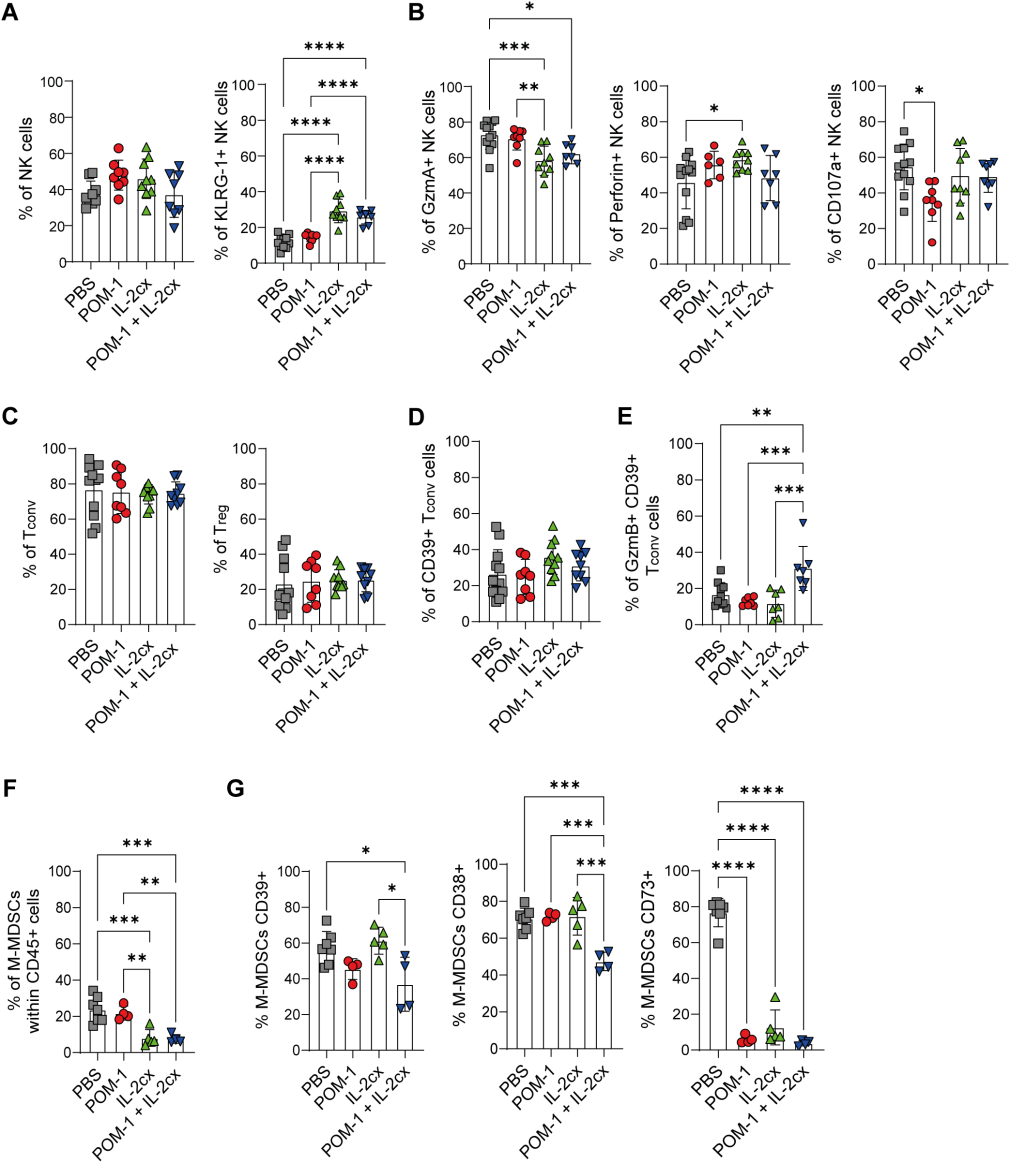
CD39 inhibition combined with IL-2cx reshapes the immune landscape of the tumor microenvironment. WT mice were injected s.c. with B16F10-OVA cells and treated with PBS (gray), POM-1 (red), IL-2/anti-IL-2 complex (IL-2cx, green), or the combination POM-1 + IL-2cx (blue). **(A)** Frequencies of T-I NK cells (left) and KLRG-1^+^ NK cells (right). **(B)** Frequencies of GzmA^+^, Perforin^+^, or CD107a^+^ T-I NK cells. **(C)** Frequencies of T-I CD4^+^ conventional T cells (Tconv left) and Treg (right). **(D)** Frequencies of CD39^+^ cells within T-I Tconv cells. **(E)** Frequencies of GzmB^+^ CD39^+^ Tconv cells. **(F)** Frequencies of T-I monocytic myeloid-derived suppressor cells (M-MDSC). **(G)** Frequencies of T-I CD39^+^, CD38^+^, or CD73^+^ expressing M-MDSCs. All data were collected on day 15 p.i. Data are presented as mean ± SD. Statistical analysis was performed using one-way ANOVA with multiple comparisons. Non-significant differences are not shown; *P ≤ 0.05; **P ≤ 0.01; ***P ≤ 0.001; ****P ≤ 0.0001.

We next evaluated the effect of the combined therapy on other immune cell populations involved in either promoting or inhibiting tumor growth, including NK cells, conventional CD4^+^ T cells (Tconv), Tregs, and myeloid cells (gating strategy in **Supplementary Figures 6A-C**). As shown in **Figure 6A**, the frequencies of NK cells did not differ among the treated groups. However, mice treated with IL-2cx or the POM-1/IL-2cx combination exhibited increased frequencies of activated NK cells (KLRG1^+^) compared to the POM-1 or control groups. Despite these phenotypic changes, we did not detect an increase in NK cell effector function, as measured by the production of GzmA, Perforin, and CD107a in mice treated with the POM-1/IL-2cx combination (**Figure 6B**).

We next examined the CD4^+^ T cell compartment and found that none of the treatments augmented the recruitment of either Tconv (CD4^+^FOXP3^-^) or Treg (CD4^+^FOXP3^+^) cells to the tumor site (**Figure 6C**). Our previous work showed that T-I CD39^+^ CD4^+^ Tconv cells exhibit cytotoxic potential (27). Therefore, we evaluated the presence of CD39-expressing Tconv cells within the TME. Although the frequency of CD39^+^ T-I CD4^+^ Tconv cells remained unchanged across treatment groups, mice receiving the POM-1/IL-2cx combined therapy showed higher expression of GzmB in this CD4^+^ T cell compartment compared to controls or monotherapy with POM-1 or IL-2cx (**Figures 6D, E**).

Finally, given the pivotal role of the myeloid compartment in tumor progression, we analyzed T-I monocytic myeloid-derived suppressor cells (M-MDSCs), total and M2-like macrophages. POM-1 treatment did not modify the frequency of T-I M-MDSCs (CD11b^+^ F4/80^-^Ly6G^-^Ly6C^+^), whereas IL-2cx significantly reduced this population compared to the control group. A similar significant decrease was observed in mice receiving the combined POM-1/IL-2cx treatment (**Figure 6F**). Interestingly, analysis of molecules associated with the immunosuppressive adenosine pathway (33) revealed that mice receiving the POM-1/IL-2cx combined treatment had a lower proportion of CD39^+^ M-MDSCs compared with the control or IL-2cx-treated groups, as well as reduced frequencies of CD38^+^ and CD73^+^ M-MDSCs compared with all treated groups or with the PBS group, respectively (**Figure 6G**). No differences were observed in the frequency of macrophages (CD11b^+^F4/80^+^Ly6G^-^Ly6C^-^) or M2-like macrophages (CD11b^+^Ly6G^-^Ly6C^-^F4/80^+^CD206^+^) (**Supplementary Figure 6D**).

Altogether, these results indicate that POM-1/IL-2cx combined therapy not only enhances the expression of cytotoxic molecules on pre-exhausted CD8^+^ T cells but also reshapes the tumor immune landscape by modulating the CD4^+^ T cell compartment and reducing the immunosuppressive profile of M-MDSCs.

## Discussion

A wide range of immunotherapeutic strategies has been developed to enhance antitumor immunity (19), and their rational combination is emerging as a promising approach to overcome resistance to ICB (34). In this study, we first investigated the impact of CD39 deficiency in tumor progression using two distinct models with differing immunogenic profiles. In MC38-bearing mice, the absence of CD39 significantly reduced tumor growth, in line with previous work and more recent study showing that anti-CD39 antibody treatment improves tumor control in comparable models (35). Li et al. (35) demonstrated that this effect depended on hematopoietic, but not non-hematopoietic, expression of CD39 and was mediated by CD8^+^ T cells and IFN-γ production. In our study, tumors from MC38-bearing WT and CD39KO mice were infiltrated by CD8^+^ T cells with an exhausted phenotype, and CD39 deficiency was associated with higher frequencies of CD8^+^ T cells co-expressing multiple iRs and expressing TOX. These findings are consistent with studies showing that anti-CD39 therapy increases IFN-γ^+^ CD8^+^ T cells co-expressing exhaustion markers (35).

Our results suggest that CD39 loss favors the accumulation of PD-1^High^ cytotoxic CD8^+^ T cells, a subset previously linked to improved survival in breast cancer patients (29, 36).

In contrast, the protective effect of CD39 deficiency observed in MC38 was not wholly reproduced in the other immunogenic B16F10-OVA model, where tumor progression was unaffected. This is consistent with reports showing that anti-CD39 treated mice fail to control tumor growth in B16F10, although tumor control can be achieved when anti-CD39 antibodies are combined with anti-PD-1 (35). Other studies, however, have shown delayed tumor growth and prolonged survival in B16F10-bearing CD39KO mice (22) as well as strong inhibition of hepatic melanoma metastases in *Cd39*-null animals (24).

These discrepancies may reflect differences in tumor antigenicity, differing inoculation protocols, and/or microbiota composition. Despite the seeming lack of tumor control in our setting, we observed that tumors from B16F10-OVA–bearing mice lacking CD39 did in fact harbor increased frequencies of both total and OVA-specific CD8^+^ T cells with a pre-exhausted PD-1^Int^ phenotype, a population that expresses low levels of TOX and high levels of TCF-1.

This observation is particularly relevant in light of studies showing that TCF-1 is crucial for optimal priming of tumor antigen-specific CD8^+^ T cells and for ICB efficacy in poorly immunogenic tumors, but dispensable in highly immunogenic settings (37). In line with this, Miller et al. (9) have reported that progenitor-exhausted CD8^+^ T cells are the subset that retains proliferative potential and responsiveness to ICB. Therefore, although CD39 deficiency does not restrain tumor growth in B16F10-OVA per se, this does shape the TME by favoring the accumulation of PD-1^Int^ TCF-1^+^ pre-exhausted CD8^+^ T cells, which may render these tumors more amenable to subsequent ICB therapy.

We then evaluated whether therapeutic CD39 inhibition in combination with IL-2cx could enhance antitumor immunity. Pharmacological inhibition of CD39 with POM-1 represents an effective approach to limit the hydrolysis of immunogenic ATP and prevent the accumulation of immunosuppressive adenosine. It has been explored as a chemotherapeutic agent in various cancers (38, 39) and shown promise as an adjunct therapy for metastatic hepatic malignancies (24), while IL-2cx formulations selectively expand CD8^+^ and NK cells, minimizing Treg activation (25, 40, 41).

In this study, POM-1 alone had minimal, non-significant impacts on tumor growth, while IL-2cx improved tumor control, and the combination was significantly more effective. In contrast, it has been reported that POM-1 suppressed experimental and spontaneous metastases in different experimental tumor models (24, 42). Our data do clearly indicate that CD39 inhibition becomes most effective when combined with therapies that enhance CD8^+^ T cell function. This interpretation is consistent with other reports showing enhanced efficacy when POM-1, anti-CD39, or IL-2cx are combined with ICB (25, 35, 42).

Several strategies have been developed to reintroduce IL-2 into clinical use (40, 41). For example, Piper et al. (43) demonstrated that radiation therapy combined with a PD-1–targeted IL-2 variant (PD1–IL2v) expanded polyfunctional CD8^+^ T cells, enhanced T cell stemness, activated NK cells, and reduced Treg frequencies in a therapy-resistant PDAC KPC-driven tumor model. These findings highlight the potential of IL-2–based strategies, particularly in combination with other immunomodulatory approaches, to enhance antitumor immunity in otherwise resistant tumor settings.

At the mechanistic level, we observed that POM-1/IL-2cx combined therapy promoted CD8^+^ T cell infiltration into tumors and increased the expression of cytotoxic molecules, particularly GzmB and Perforin, within pre-exhausted PD-1^Int^ CD8^+^ T cells despite reduced proliferative capacity. This suggests a qualitative shift toward enhanced cytotoxicity, consistent with the role of pre-exhausted CD8^+^ T cells as a progenitor pool with potential for effector differentiation (9, 44). In contrast, PD-1^High^ CD8^+^ T cells did not upregulate cytotoxic molecules, highlighting their limited responsiveness to this intervention (9). The pre-exhausted CD8^+^ T cells expressing GzmB or Perforin were absent in dLNs, consistent with previous reports that, in mouse tumor models, tumor-antigen-specific CD8^+^ T cells become activated but fail to express effector molecules such as GzmB (45).

Moreover, in contrast to what has been reported (25), we observed that, regardless of the treatment strategy used, treated mice displayed a decreased proportion of OVA-specific CD8^+^ T cells compared with controls. However, these results are consistent with findings in patients with lung cancer (46) and basal cell carcinoma (47), where αPD-1 therapy leads to the expansion of LiT CD8^+^ T cell clonotypes that differ from those present in the tumor before treatment.

Our findings support a model where CD39 inhibition and IL-2cx synergize to boost the effector capacity of pre-exhausted CD8^+^ T cells, thereby contributing to tumor control.

Other immune compartments were also affected. IL-2cx, alone or combined with POM-1, increased the frequency of activated NK cells (KLRG1^+^), but without a corresponding rise in cytotoxic molecule expression. This is consistent with previous reports showing that IL-2cx promotes tumor control by expanding T-I NK cells and increasing the proportion of KLRG1^+^ NK cells (25). Notably, POM-1 did not further enhance IL-2cx-mediated NK cell activation in our model. This contrasts with reports showing strong anti-metastatic effects of POM-1 in combination with ICB, BRAFi/MEKi, or IL-2, as well as studies demonstrating reduced lung metastases through increased NK cell numbers and IFN-γ production (42). These discrepancies may reflect differences in experimental models, since NK cell–mediated effects of POM-1 have been mainly described in metastatic settings (24, 42). In the CD4^+^ T cell compartment, neither IL-2cx nor its combination with POM-1 increased Tconv or Treg infiltration. However, POM-1/IL-2cx combined therapy enhanced GzmB expression in CD39^+^ CD4^+^ Tconv cells. This subset was characterized by Bossio et al. (27) as cytotoxic and exhausted with expression of multiple iRs, IFN-γ, GzmB, Perforin, and CD107a. Notably, high CD4 and ENTPD1 (CD39) expression in human tumor tissues has been linked to improved overall survival in breast cancer patients, supporting previous reports (48, 49) that highlighted the contribution of cytotoxic CD4^+^ T cells to antitumor immunity. Finally, POM-1/IL-2cx combined therapy reduced the expression of CD39, CD38, and CD73 on M-MDSCs, potentially decreasing adenosine production in the TME. This aligns with studies showing that myeloid-expressed CD39 is essential for metastasis control by anti-CD39 (50), and with our other work demonstrating that afucosylated anti-CD39 antibodies also deplete CD39^high^ tumor-associated macrophages and endothelial cells, impairing angiogenesis (23).

In conclusion, our findings demonstrate that combining blockade of CD39 ecto-enzymatic activity with IL-2–based therapies boosts anti-tumor effects. This approach remodels both lymphoid and myeloid compartments, promotes the cytotoxic differentiation of pre-exhausted CD8^+^ T cells, enhances cytotoxic CD4^+^ T cell activity, and decreases the expression of certain purinergic molecules in myeloid cells, which may otherwise mediate immunosuppression by this population. These data provide a strong rationale for exploring CD39-targeted strategies in combination with IL-2–based therapies and ICB in translational clinical studies.

## Data Availability

The original contributions presented in the study are included in the article/**Supplementary Material**. Further inquiries can be directed to the corresponding author.

## Supplementary material

Supplementary Figure 1 Gating strategy. **(A)** Representative contour plots showing CD39 expression on T-I live cells from WT (gray) and CD39KO (orange) mice injected s.c. with MC38 (left) or B16F10-OVA (right) tumor cells. **(B)** Gating strategy used to identify CD8^+^ T cells, conventional CD4^+^ T cells, (Tconv), regulatory CD4^+^ T cells (Treg), B cells, monocytes (Mo), and other CD11b^+^ cells in both WT and CD39KO tumor-bearing mice. **(C)** Frequencies of CD8^+^ T cells co-expressing 4 iRs from WT (gray) and CD39KO (orange) MC38 tumor-bearing mice on 17 d.p.i. **(D)** Bar graphs showing the frequencies of TOX^+^ and TCF-1^+^ expressing T-I CD8^+^ T cells from WT (gray) and CD39KO (orange) MC38 tumor-bearing bearing on 17 d.p.i. Results were obtained from at least 2 independent experiments. Data are presented as mean ± SD. Statistical analysis was performed using unpaired Student’s *t*-test. ns: not significant; **P ≤ 0.01; *****P* ≤ 0.0001.

Supplementary Figure 2 Phenotypic profiling of PD-1- expressing CD8^+^ T cells of MC38 tumors. WT (gray) and CD39KO (orange) mice were injected s.c. with MC38 tumor cells **(A)** Representative contour plots and frequencies of TIM-3^+^ cells within PD-1^Neg^, PD-1^Int^, and PD-1^High^ T-I CD8^+^ T cells. **(B)** TOX expression (MFI) within PD-1^Neg^, PD-1^Int^, and PD-1^High^ T-I CD8^+^ T cells. UC: unstained control **(C)** TCF-1 expression (MFI) within PD-1^Neg^, PD-1^Int^, and PD-1^High^ T-I CD8^+^ T cells. UC: unstained control. **(D)** Frequencies of GzmB^+^ or Perforin^+^ cells within PD-1^Neg^, PD-1^Int^, and PD-1^High^ CD8^+^ T cells. Data was collected on day 17 p.i. Results were obtained from at least 2 independent experiments. Data is presented as mean ± SD. Statistical analysis was performed using one-way ANOVA with multiple comparisons test. ns: not significant; **P* ≤ 0.05; ***P* ≤ 0.01; *****P* ≤ 0.0001.

Supplementary Figure 3 Functional and phenotypic characterization of PD-1-expressing CD8^+^ T cells of B16F10-OVA tumors. WT (gray) and CD39KO (orange) mice were injected s.c. with B16F10-OVA tumor cells. **(A)** Frequencies of T-I CD45^+^ cells. **(B)** Frequencies of T-I CD8^+^ T cells co-expressing 3 iRs (PD-L1, 2B4 and TIM-3) within PD-1^Neg^, PD-1^Int^, and PD-1^High^ T-I CD8^+^ T cells. **(C)** Frequencies of TIM-3^+^ cells, **(D)** Frequencies of TOX^+^ cells and **(E)** Frequencies of TCF-1^+^ cells within PD-1^Neg^, PD-1^Int^, and PD-1^High^ T-I CD8^+^ T cells. **(F)** Representative contour plot and frequencies of IFN-γ^+^ cells within PD-1^Neg^, PD-1^Int^, and PD-1^High^ T-I CD8^+^ T cells at 15 d.p.i. **(G)** Representative contour plots and frequencies of CD107a^+^, GzmB^+^, and Perforin^+^ cells within PD-1^Neg^, PD-1^Int^, and PD-1^High^ CD8^+^ T cells. Data is presented as mean ± SD. Statistical significance was assessed using unpaired Student’s *t*-test **(A, B)** or one-way ANOVA with Sidak’s multiple comparisons test **(C–H)**. ns: not significant; **P* ≤ 0.05; ***P* ≤ 0.01; ****P* ≤ 0.001; *****P* ≤ 0.0001.

Supplementary Figure 4 Antitumor effects of CD39 inhibition and IL-2cx treatment. WT mice were injected s.c. with B16F10-OVA tumor cells and treated with PBS (gray), POM-1 (red), IL-2cx, (green), or POM-1 + IL-2cx (blue), and analyzed at day 15 p.i. **(A)** (Left) Individual tumor growth curves from 7–15 dpi in treated mice. (Right) Bar graph showing tumor volume comparisons across treatment groups at different d.p.i. **(B)** Representative contour plots show T-I OVA-specific CD8^+^ T cells for each treatment condition. **(C)** Frequencies of PD-1^High^ cells (Left) and TOX^+^ cells (Right) within OVA-specific T-I CD8^+^ T cells. Representative data from one of four independent experiments. **(D)** Frequencies of CD45^+^ leukocytes and CD8^+^ T cells in dLNs. **(E)** Frequencies of GzmB^+^, Perforin^+^, CD107a^+^, and IFN-γ^+^ CD8^+^ T cells in dLNs. Data is presented as mean ± SD. Statistical analysis was performed using one-way ANOVA with multiple comparisons test. Non-significant differences are not shown; **P* ≤ 0.05; ***P* ≤ 0.01; ****P* ≤ 0.001; *****P* ≤ 0.0001.

Supplementary Figure 5 Functional profiling of PD-1^High^ CD8^+^ T cells. WT mice were injected s.c. with B16F10-OVA cells and treated with PBS (gray), POM-1 (red), IL-2cx, (green), or POM-1 + IL-2cx (blue), and analyzed at day 15 p.i. **(A)** Frequencies of GzmB^+^, Perforin^+^, CD107a^+^, and IFN-γ^+^ cells within T-I PD-1^High^ CD8^+^ T cells. Data is presented as mean ± SD. Statistical analysis was performed using one-way ANOVA with multiple comparisons test. Non-significant differences are not shown; **P* ≤ 0.05; ***P* ≤ 0.01; *****P* ≤ 0.0001.

Supplementary Figure 6 Gating strategies and analysis of immune cells subsets in treated tumor-bearing mice. WT mice injected s.c. with B16F10-OVA cells and treated with PBS (gray), POM-1 (red), IL-2cx, (green), or POM-1 + IL-2cx (blue), and analyzed at day 15 p.i. **(A)** Gating strategy used to identify T-I NK, **(B)** T-I conventional CD4^+^ T cells (Tconv), Treg cells **(C)** Gating strategy used to identify T-I M-MDSCs and M2-like macrophages in treated groups. **(D)** Frequencies of T-I Macrophages within CD45^+^ cells (left) and T-I M2 Macrophages within F4/80^+^Ly6G^-^Ly6C^-^ (right) in treated groups. Data is presented as mean ± SD. Statistical analysis was performed using one-way ANOVA with multiple comparisons test. Non-significant differences are not shown.
