# Supplementary figures and images for: Targeting CD39 in combination with IL-2/anti-IL-2 complexes enhances cytotoxic immunity and limits tumor progression
Source: Front Immunol. 2026 Jan 26;17:1730342. doi: 10.3389/fimmu.2026.1730342 (PMC12883794; doi:10.3389/fimmu.2026.1730342)

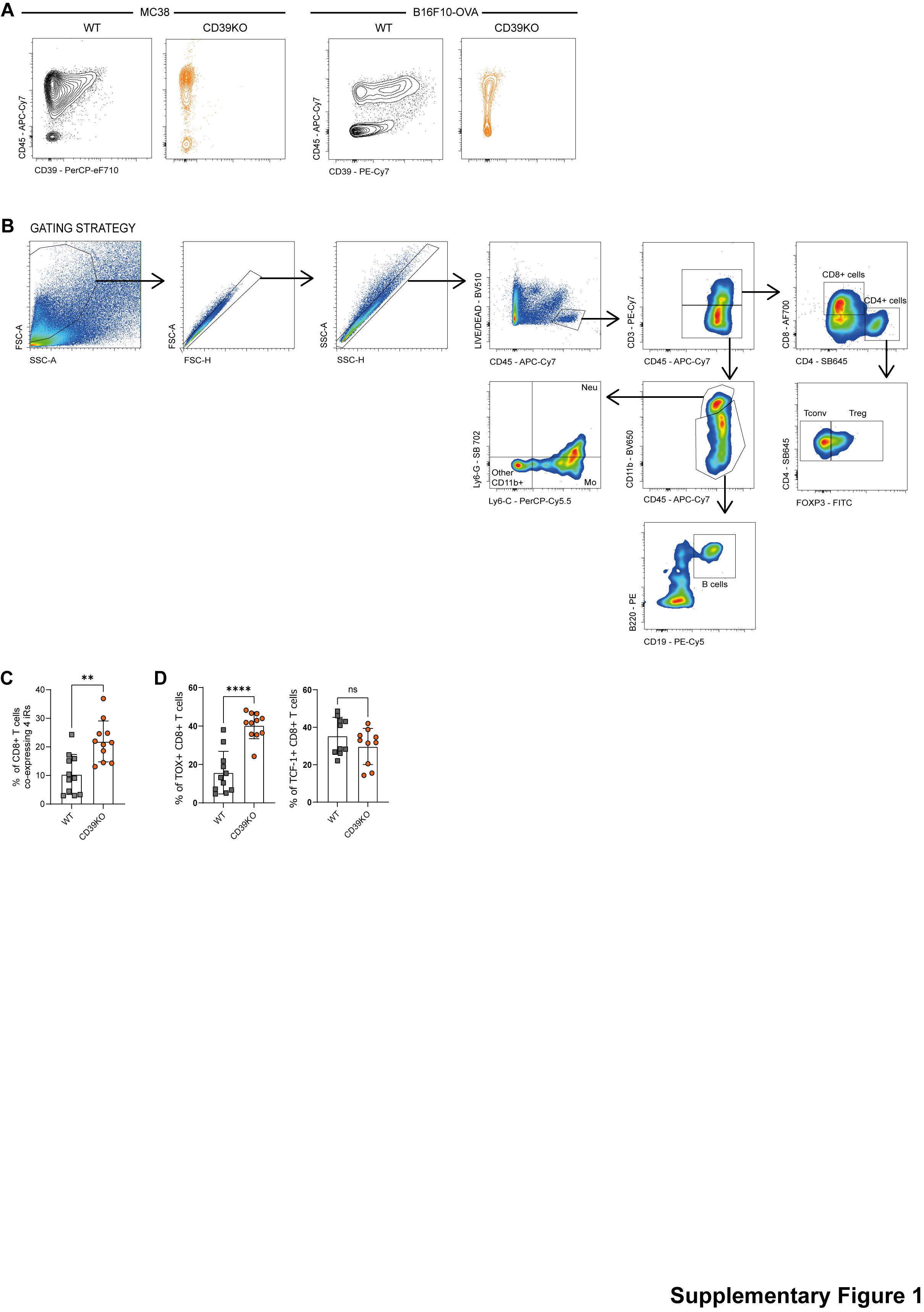

Supplement: Supplementary Figure 1 — Gating strategy. (A) Representative contour plots showing CD39 expression on T-I live cells from WT (gray) and CD39KO (orange) mice injected s.c. with MC38 (left) or B16F10-OVA (right) tumor cells. (B) Gating strategy used to identify CD8+ T cells, conventional CD4+ T cells, (Tconv), regulatory CD4+ T cells (Treg), B cells, monocytes (Mo), and other CD11b+ cells in both WT and CD39KO tumor-bearing mice. (C) Frequencies of CD8+ T cells co-expressing 4 iRs from WT (gray) and CD39KO (orange) MC38 tumor-bearing mice on 17 d.p.i. (D) Bar graphs showing the frequencies of TOX+ and TCF-1+ expressing T-I CD8+ T cells from WT (gray) and CD39KO (orange) MC38 tumor-bearing bearing on 17 d.p.i. Results were obtained from at least 2 independent experiments. Data are presented as mean ± SD. Statistical analysis was performed using unpaired Student’s t-test. ns: not significant; **P ≤ 0.01; ****P ≤ 0.0001. [file Image1.tif]

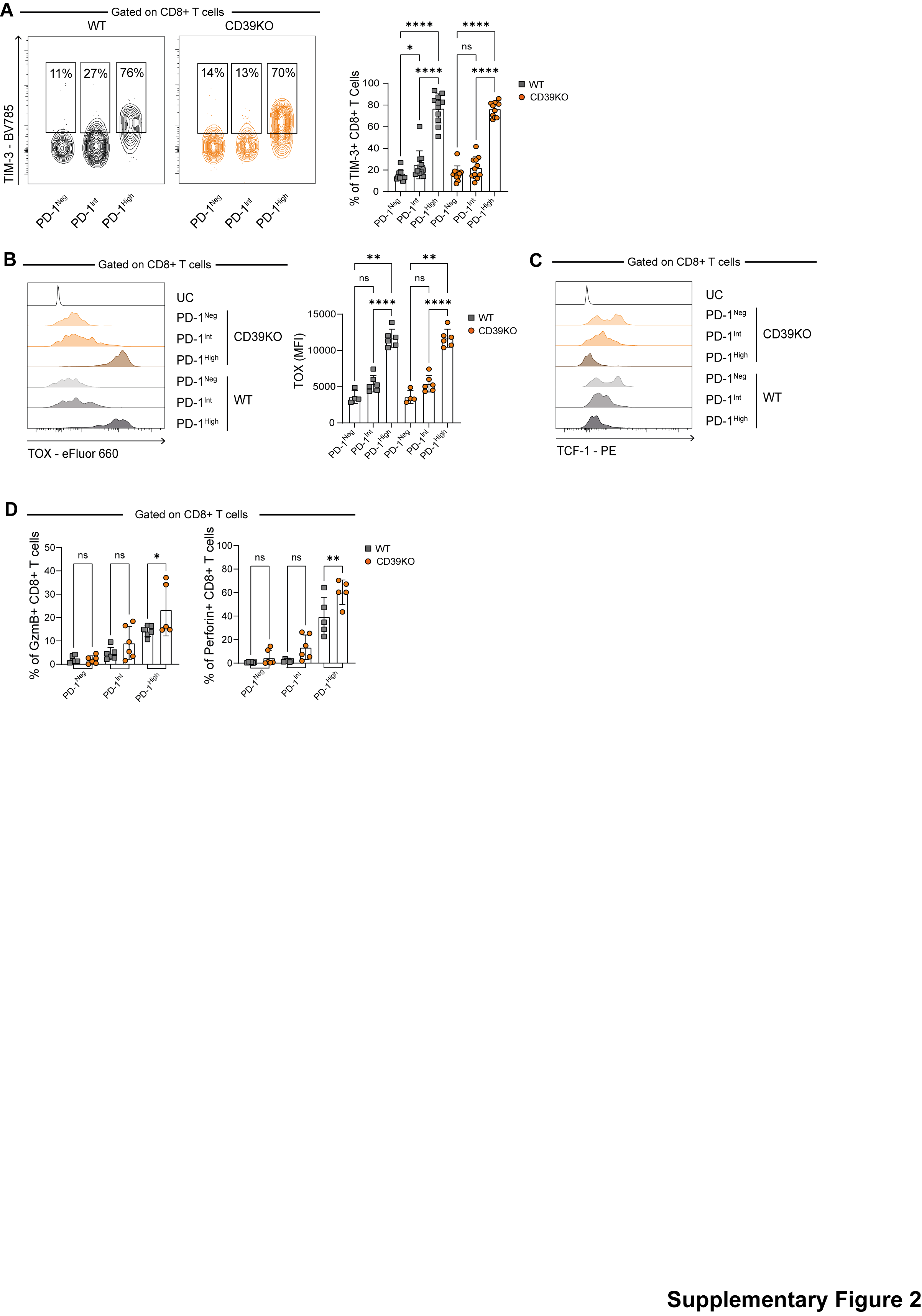

Supplement: Supplementary Figure 2 — Phenotypic profiling of PD-1- expressing CD8+ T cells of MC38 tumors. WT (gray) and CD39KO (orange) mice were injected s.c. with MC38 tumor cells (A) Representative contour plots and frequencies of TIM-3+ cells within PD-1Neg, PD-1Int, and PD-1High T-I CD8+ T cells. (B) TOX expression (MFI) within PD-1Neg, PD-1Int, and PD-1High T-I CD8+ T cells. UC: unstained control (C) TCF-1 expression (MFI) within PD-1Neg, PD-1Int, and PD-1High T-I CD8+ T cells. UC: unstained control. (D) Frequencies of GzmB+ or Perforin+ cells within PD-1Neg, PD-1Int, and PD-1High CD8+ T cells. Data was collected on day 17 p.i. Results were obtained from at least 2 independent experiments. Data is presented as mean ± SD. Statistical analysis was performed using one-way ANOVA with multiple comparisons test. ns: not significant; *P ≤ 0.05; **P ≤ 0.01; ****P ≤ 0.0001. [file Image2.tif]

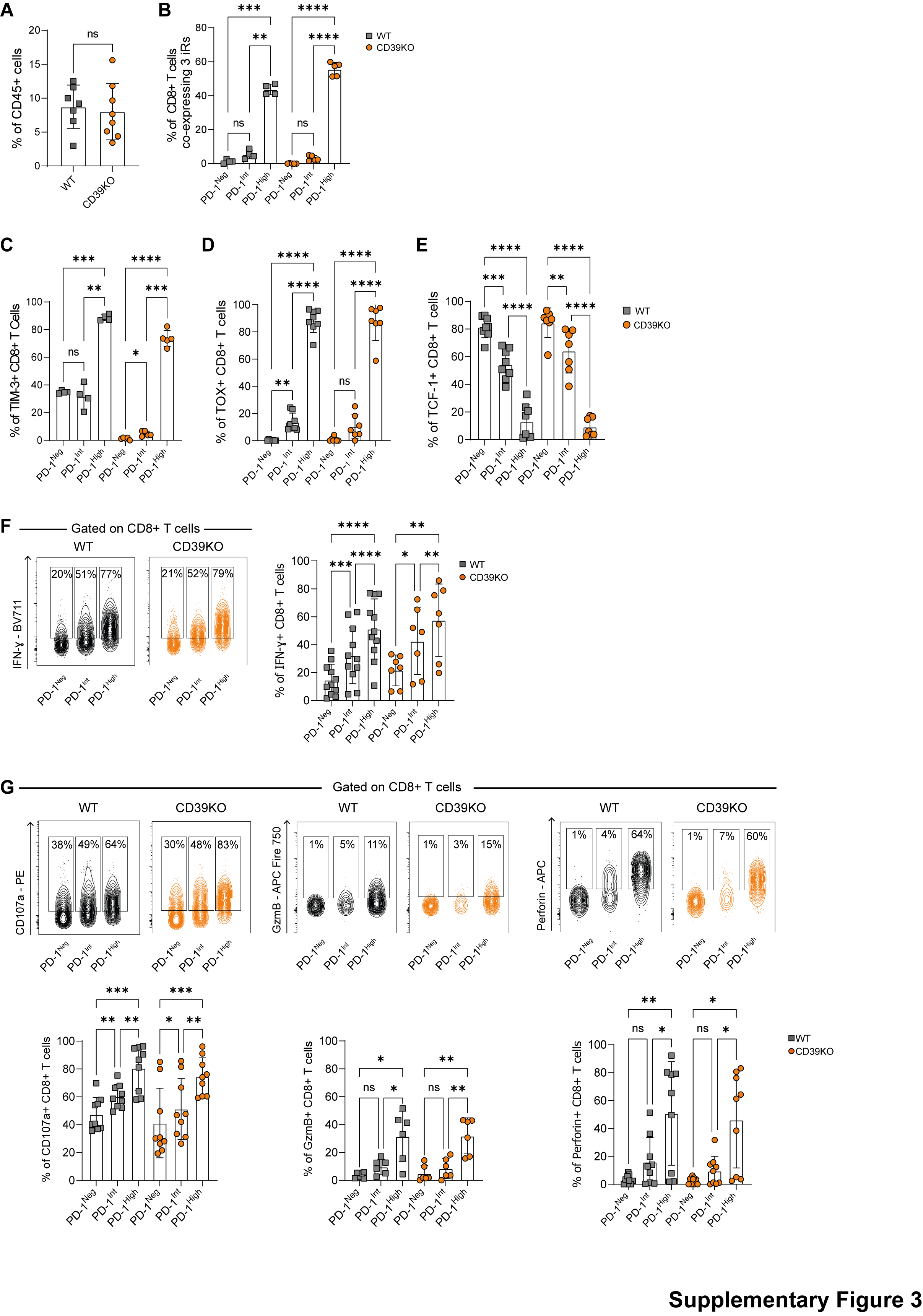

Supplement: Supplementary Figure 3 — Functional and phenotypic characterization of PD-1-expressing CD8+ T cells of B16F10-OVA tumors. WT (gray) and CD39KO (orange) mice were injected s.c. with B16F10-OVA tumor cells. (A) Frequencies of T-I CD45+ cells. (B) Frequencies of T-I CD8+ T cells co-expressing 3 iRs (PD-L1, 2B4 and TIM-3) within PD-1Neg, PD-1Int, and PD-1High T-I CD8+ T cells. (C) Frequencies of TIM-3+ cells, (D) Frequencies of TOX+ cells and (E) Frequencies of TCF-1+ cells within PD-1Neg, PD-1Int, and PD-1High T-I CD8+ T cells. (F) Representative contour plot and frequencies of IFN-γ+ cells within PD-1Neg, PD-1Int, and PD-1High T-I CD8+ T cells at 15 d.p.i. (G) Representative contour plots and frequencies of CD107a+, GzmB+, and Perforin+ cells within PD-1Neg, PD-1Int, and PD-1High CD8+ T cells. Data is presented as mean ± SD. Statistical significance was assessed using unpaired Student’s t-test (A, B) or one-way ANOVA with Sidak’s multiple comparisons test (C–H). ns: not significant; *P ≤ 0.05; **P ≤ 0.01; ***P ≤ 0.001; ****P ≤ 0.0001. [file Image3.tif]

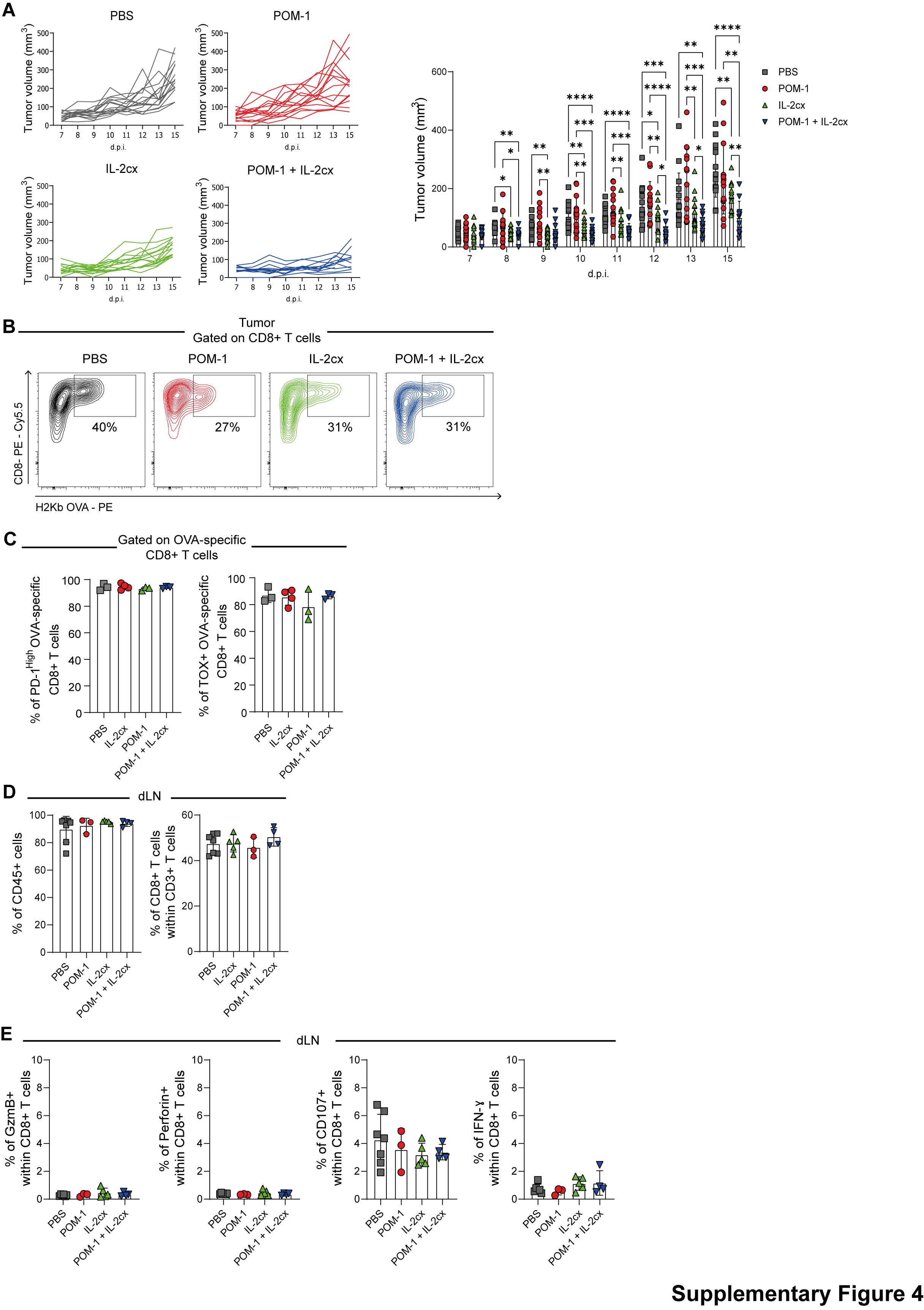

Supplement: Supplementary Figure 4 — Antitumor effects of CD39 inhibition and IL-2cx treatment. WT mice were injected s.c. with B16F10-OVA tumor cells and treated with PBS (gray), POM-1 (red), IL-2cx, (green), or POM-1 + IL-2cx (blue), and analyzed at day 15 p.i. (A) (Left) Individual tumor growth curves from 7–15 dpi in treated mice. (Right) Bar graph showing tumor volume comparisons across treatment groups at different d.p.i. (B) Representative contour plots show T-I OVA-specific CD8+ T cells for each treatment condition. (C) Frequencies of PD-1High cells (Left) and TOX+ cells (Right) within OVA-specific T-I CD8+ T cells. Representative data from one of four independent experiments. (D) Frequencies of CD45+ leukocytes and CD8+ T cells in dLNs. (E) Frequencies of GzmB+, Perforin+, CD107a+, and IFN-γ+ CD8+ T cells in dLNs. Data is presented as mean ± SD. Statistical analysis was performed using one-way ANOVA with multiple comparisons test. Non-significant differences are not shown; *P ≤ 0.05; **P ≤ 0.01; ***P ≤ 0.001; ****P ≤ 0.0001. [file Image4.tif]

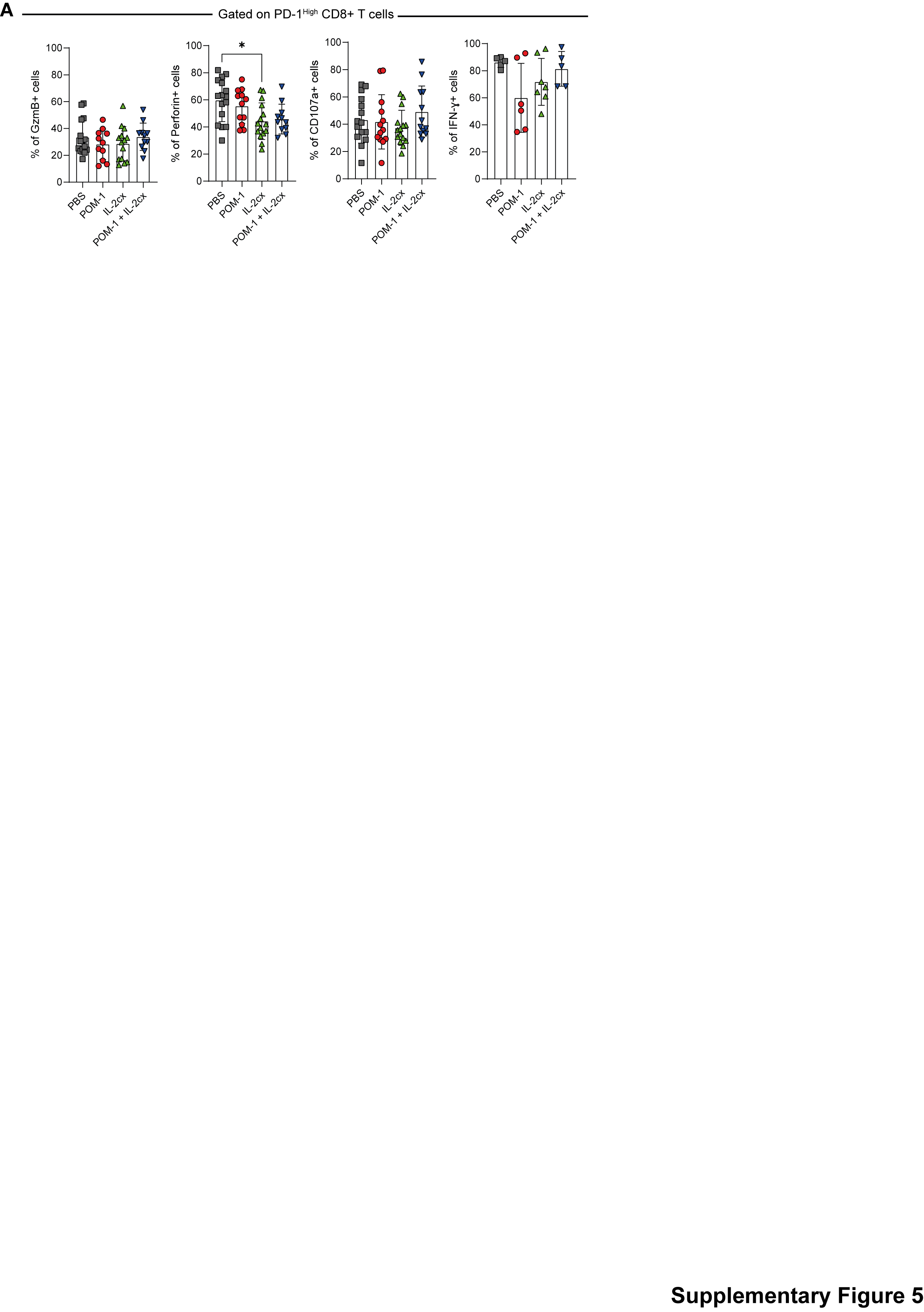

Supplement: Supplementary Figure 5 — Functional profiling of PD-1High CD8+ T cells. WT mice were injected s.c. with B16F10-OVA cells and treated with PBS (gray), POM-1 (red), IL-2cx, (green), or POM-1 + IL-2cx (blue), and analyzed at day 15 p.i. (A) Frequencies of GzmB+, Perforin+, CD107a+, and IFN-γ+ cells within T-I PD-1High CD8+ T cells. Data is presented as mean ± SD. Statistical analysis was performed using one-way ANOVA with multiple comparisons test. Non-significant differences are not shown; *P ≤ 0.05; **P ≤ 0.01; ****P ≤ 0.0001. [file Image5.tif]

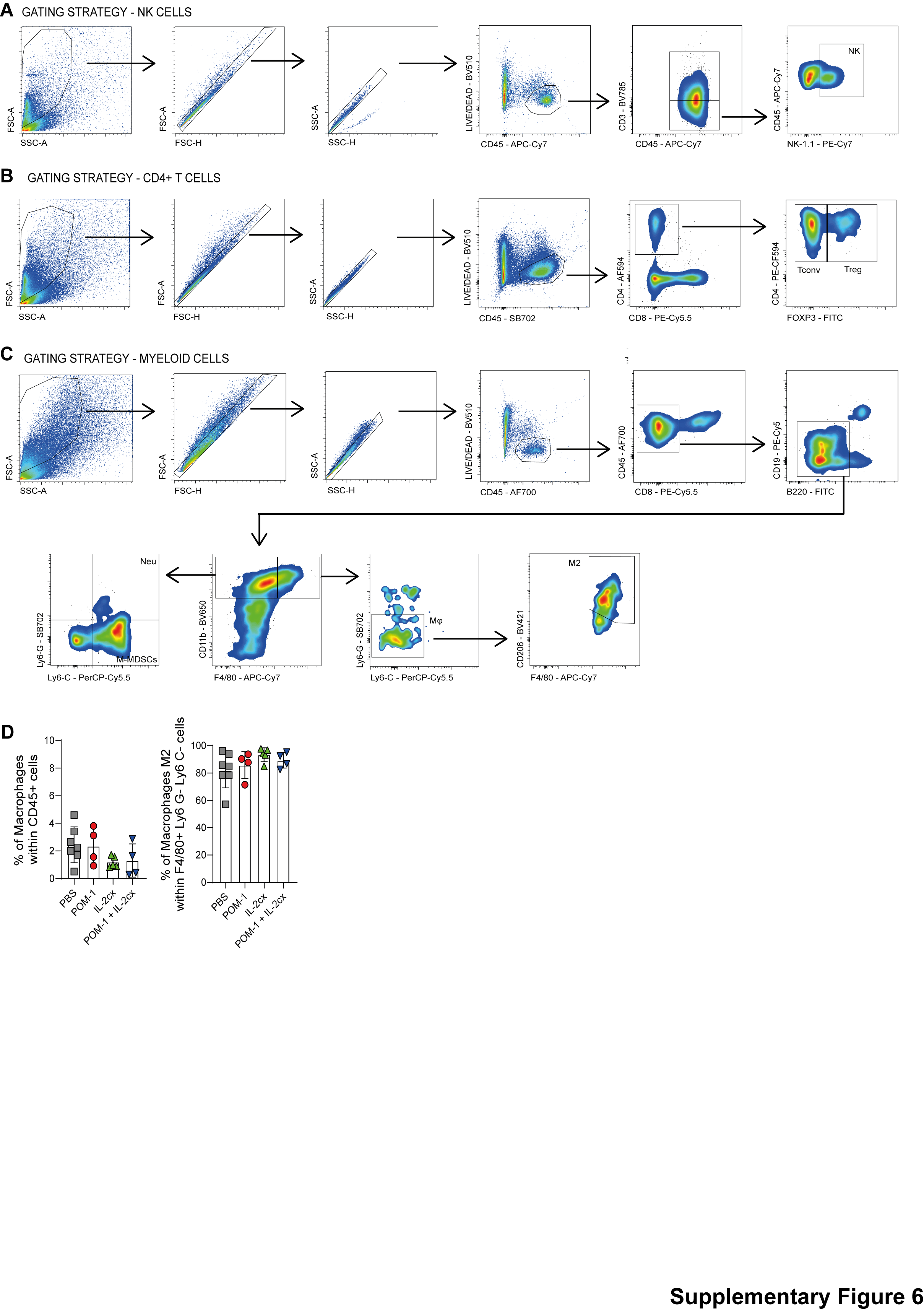

Supplement: Supplementary Figure 6 — Gating strategies and analysis of immune cells subsets in treated tumor-bearing mice. WT mice injected s.c. with B16F10-OVA cells and treated with PBS (gray), POM-1 (red), IL-2cx, (green), or POM-1 + IL-2cx (blue), and analyzed at day 15 p.i. (A) Gating strategy used to identify T-I NK, (B) T-I conventional CD4+ T cells (Tconv), Treg cells (C) Gating strategy used to identify T-I M-MDSCs and M2-like macrophages in treated groups. (D) Frequencies of T-I Macrophages within CD45+ cells (left) and T-I M2 Macrophages within F4/80+Ly6G-Ly6C- (right) in treated groups. Data is presented as mean ± SD. Statistical analysis was performed using one-way ANOVA with multiple comparisons test. Non-significant differences are not shown. [file Image6.tif]
