# Supplementary material for: Targeting CD39 in combination with IL-2/anti-IL-2 complexes enhances cytotoxic immunity and limits tumor progression
Source: Front Immunol. 2026 Jan 26;17:1730342. doi: 10.3389/fimmu.2026.1730342 (PMC12883794; doi:10.3389/fimmu.2026.1730342)
Supplement: Supplementary file 7 [file DataSheet1.pdf]

Supplementary Table 1

ANTIGEN	FLUOROCROME	CLON	BRAND
LIVE/DEAD Fixable Violet Dead Cell Stain Kit			Invitrogen
CD45	AF700	30-F11	Thermofisher
CD45	Super Bright 702	30-F11	Invitrogen
CD45	APC-Cy7	30-F11	BD
CD8	FITC	53-6.7	eBioscience
CD8	AF700	53-6.7	eBioscience
CD8	PE-Cy5.5	53-6.7	eBioscience
CD3	BV785	145-2c11	Biolegend
CD3	PE-Cy7	145-2C11	eBioscience
CD4	Super Bright 645	GK1.5	Invitrogen
CD4	PE-CF 594	RM4-5	BD
FOXP3	FITC	FJK-16s	eBioscience
CD11b	BV650	M1/70	eBioscience
Ly6-C	PerCp-Cy5.5	HK1.4	eBioscience
Ly6-G	Super Bright 702	1AB	Biolegend
CD19	PE-Cy5	eBio1D3	eBioscience
B220	PE	RA3-6B2	Biolegend
B220	FITC	RA3-6B2	Biolegend
NK1.1	PE-Cy7	PK136	Biolegend
CD206	BV421	C068C2	Biolegend
F4-80	APC-Cy7	BM8	Biolegend
DEXTRAMERO H2Kb OVA	PE		Immudex
CD39	PerCp-eFluor.710	24DMS1	eBioscience
CD39	PE-Cy7	24DMS1	eBioscience
CD44	PE-Cy5	IM7	BD
CD62L	SB600	mel-14	eBioscience
LAG-3	PerCP-Cy5.5	C9B7W	Biolegend
PD-1	BV421	29F.1A12	Biolegend
PD-1	PE	RMP1-30	eBioscience
2B4	PE	eBio244F4	eBioscience
PD-L1	PE-Cy7	MIH5	Invitrogen
TIM-3	BV785	RMT3-23	Biolegend
TIM-3	APC	RMT3-23	Biolegend
Eomes	PE-eFluor610	Dan11mag	Invitrogen
T-bet	PE-Cy7	4B10	Invitrogen
IRF4	PerCP-eFluor 710	3 E4	eBioscience
TOX	eFluor 660	TXRX10	Invitrogen
TCF-1	PE	S33-966	BD
IFN-ɣ	APC	XMG1.2	BD
IFN-ɣ	BV711	XMG1.2	BD
IFN-ɣ	BV421	XMG1.2	Biolegend
CD107a	PE	1D4B	Biolegend
Perforin	APC	S16009A	Biolegend
GzmB	APC/Fire 750	QA16A02	Biolegend
